# Fibrin Networks Regulate Protein Transport during Thrombus Development

**DOI:** 10.1371/journal.pcbi.1003095

**Published:** 2013-06-13

**Authors:** Oleg V. Kim, Zhiliang Xu, Elliot D. Rosen, Mark S. Alber

**Affiliations:** 1 Department of Applied and Computational Mathematics and Statistics, University of Notre Dame, South Bend, Indiana, United States of America; 2 Department of Medical and Molecular Genetics, Indiana University School of Medicine, Indianapolis, Indiana, United States of America; 3 Department of Medicine, Indiana University School of Medicine, Indianapolis, Indiana, United States of America; University of California San Diego, United States of America

## Abstract

Thromboembolic disease is a leading cause of morbidity and mortality worldwide. In the last several years there have been a number of studies attempting to identify mechanisms that stop thrombus growth. This paper identifies a novel mechanism related to formation of a fibrin cap. In particular, protein transport through a fibrin network, an important component of a thrombus, was studied by integrating experiments with model simulations. The network permeability and the protein diffusivity were shown to be important factors determining the transport of proteins through the fibrin network. Our previous *in vivo* studies in mice have shown that stabilized non-occluding thrombi are covered by a fibrin network (‘fibrin cap’). Model simulations, calibrated using experiments in microfluidic devices and accounting for the permeable structure of the fibrin cap, demonstrated that thrombin generated inside the thrombus was washed downstream through the fibrin network, thus limiting exposure of platelets on the thrombus surface to thrombin. Moreover, by restricting the approach of resting platelets in the flowing blood to the thrombus core, the fibrin cap impaired platelets from reaching regions of high thrombin concentration necessary for platelet activation and limited thrombus growth. The formation of a fibrin cap prevents small thrombi that frequently develop in the absence of major injury in the 60000 km of vessels in the body from developing into life threatening events.

## Author Summary

To restrict the loss of blood following rupture of blood vessels, the human body rapidly forms a clot consisting mainly of platelets and fibrin. However, to prevent formation of a pathological clot within vessels (thrombus) as a result of vessel damage or dysfunction, the response must be regulated, and clot formation must be limited. Our previous studies demonstrated that as a laser-induced thrombus stabilized in mice, the ratio of fibrin to platelets at the thrombus surface increased significantly. Stabilized non-occluding thrombi were observed to be covered by a fibrin network (‘fibrin cap’). In the present work the role of the fibrin network in protein transport is examined by integrating experiments in microfluidic devices with the hemodynamic thrombus model. The study reveals permeability of the fibrin network and protein diffusivity to be important factors determining the transport of blood proteins inside the thrombus. It is shown that the fibrin network does not dramatically limit the diffusion of thrombin but impairs flowing platelets in blood from reaching regions of high thrombin concentration thus, reducing the probability they are activated and stably integrated into the thrombus. This novel, counter-intuitive mechanism suggests that a fibrin network formed at early stages of thrombus initiation can prevent normally asymptomatic thrombi from developing into pathological clots.

## Introduction

Damage or inflammation to the blood vessel wall initiates the development of intravascular clots (thrombi). Uncontrolled growth of thrombi can result in occlusion of the blood vessel, starving tissues in the flow field of nutrients and oxygen. Furthermore, clot fragments washed away from the thrombus (emboli) may lodge in the vasculature of the lungs and brain causing life threatening conditions such as pulmonary embolism and ischemic stroke. Thromboembolic disease is a major biomedical problem with 900,000 cases per year in the USA leading to 300,000 deaths [1].

**Figure 1:**
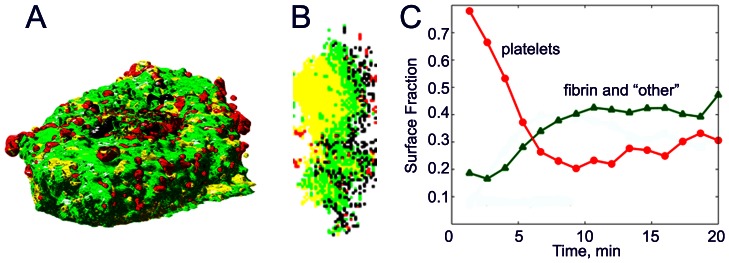
Composition of developing thrombi obtained from vertical stacks of images collected by multiphoton microscopy of laser induced injuries in mesenteric veins of a mice. 3D image reconstruction of a late stage thrombus is shown in (a) (luminal view) and (b) cross section in a vertical plane (wall is on the left, lumen is on the right). Regions composed mostly of platelets are red, mostly of fibrin - green, composed of platelets and fibrin -yellow; and regions excluding plasma, fibrin and platelets (other material, cells) - black. (c) Shows evolution of the thrombus composition as it stabilizes. Stabilization is associated with decreasing amounts of platelets and increasing amounts of fibrin on the surface.

We have reported [2]–[5] that following laser-induced injury to a few cells lining the vessel wall small non-occlusive thrombi initially grow rapidly by accumulating platelets. After several minutes, growth ceases and the thrombi stabilize. The cessation of growth occurs as the surface composition of the developing thrombus changes. While actively growing during the first few minutes following injury the surface is composed primarily of platelets. As the thrombus stabilizes the surface composition changes to one composed primarily of a fibrin network containing cellular and platelet clusters (Figure 1).

We previously developed multi-scale models of clot formation taking into account all main components including hemodynamics, molecular signaling leading to platelet activation and coagulation biochemical reactions taking place in blood and on surfaces of platelets [4], [6]. A combination of experimental approaches, image analysis and multi-scale modeling was used for formulating a novel biological hypothesis of a fibrin cap being capable of stopping blood clot growth by interfering with the transport of thrombin to the thrombus surface. Thrombin is a potent platelet activator and is required for fibrin generation [4], [7], [8]. By interfering with thrombin transport to the thrombus surface, we hypothesize the fibrin cap can limit further growth.

Because thrombin is primarily generated by coagulation reactions concentrated on the surface of activated platelets in the thrombus [9], it is important to study the movement of thrombin through the fibrin network to determine if the network limits the distribution of thrombin to affect thrombus growth. Conceivably, the fibrin network forms a diffusion and permeability barrier and prevents the transport of thrombin to the thrombus surface.

This paper examines this hypothesis by studying in detail protein advection and diffusion to describe thrombin transport through fibrin networks (fibrin cap). It integrates computational and experimental analysis to show how the formation of a fibrin network limits thrombus growth. Network permeability and protein diffusion are the important factors determining the transport of proteins through the fibrin network of thrombi. We report on the experimental measurements of these factors and the development of a thrombus hemodynamics model incorporating them. We show that networks formed under physiological concentrations of fibrinogen and thrombin do not appreciably affect the diffusion of thrombin indicating the fibrin network does not provide a diffusion barrier. However, it is demonstrated that in permeable fibrin network, thrombin generated by platelets is rapidly washed downstream by advection. Only a thin band upstream and above the source of thrombin maintains an appreciable thrombin concentration, similar to the distribution expected in the absence of a fibrin network. However, the pore size in a newly formed sparse network is small enough to exclude resting platelets in flowing blood from approaching the thrombus core. The resting platelets in flowing blood contacting the thrombus therefore do not reach regions with sufficiently high thrombin concentration necessary for their activation and stable incorporation into the developing thrombus. Thus, the development of a sparse, permeable fibrin network on the thrombus surface may provide a mechanism to limit continued growth of small thrombi. The network does not provide a transport barrier for thrombin but rather a shield blocking resting platelets from approaching sites of thrombin generation. Our results identify a novel mechanism and reinforces the concept that the spatial separation of sites of factor activation and factor activity are important to understanding thrombogenesis. The suggested mechanism may prevent small thrombi, which frequently form as a result of activation or damage of a few endothelial cells, from growing into symptomatic occlusive clots.

## Results/Discussion

**Figure 2:**
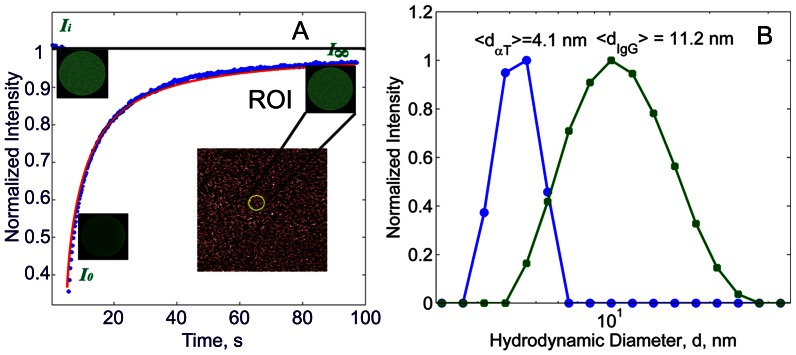
A: FRAP microscopy of fluorescently labeled Fab fragments of IgG. For a typical FRAP experiment performed on a fibrin network, the regions of interest (ROIs, radius of 27 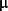
m) before, immediately after photobleaching, and after 98 s are shown (green circle). Normalized fluorescence recovery for the corresponding ROIs and their fits according to a model (Equation (7), ) are shown by symbols and a line, respectively. Fluorescence intensity values before (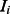) and after (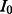) photobleaching, and at the end of the experiment (I_∞_), are shown. For each sample, FRAP measurements were repeated 3 times in 8 different locations over the sample and the arithmetic mean of the intensity curves was taken. B: Hydrodynamic diameter measurements by dynamic light scattering: circles denote 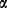-thrombin and squares denote Fab IgG.

Since thrombin is critical for continued thrombus growth, we developed a thrombus hemodynamics model to compute the thrombin concentration at different positions in the thrombus. The model assumes thrombin is generated on the surface of activated platelets in the thrombus core and is transported through the fibrin network that surrounds the core both by advective flow of fluid permeating through the fibrin network and by diffusion through the fibrin gel. To calibrate the model, we experimentally determined fibrin clot permeability and thrombin diffusion rates through a fibrin network. Permeability was determined by perfusing liquid through fibrin gels formed with a physiological range of fibrin concentrations (0.5 to 4.0 mg/ml). Protein diffusion rates were determined using Fluorescence Recovery after Photobleaching (FRAP) [10] protocols (Figure 2A). In addition, we also examined the diffusion rates of larger molecules, Fab IgG (MW = 50 kDa), in fibrin networks to assess the effect of the protein size on its diffusion. The experimentally determined measurements of permeability, protein diffusivity and the fibrin network density were then used for predictive analysis in the thrombus hemodynamic model.

### Protein Size Measurements

Before quantifying the transport of proteins in fibrin networks, protein diameter distributions were measured using Dynamic Light Scattering (DLS) (Figure 2B). The mean hydrodynamic diameter 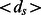 of thrombin and Fab IgG solutes were found to be 4.1 nm and 11.2 nm, respectively. These are in a remarkable agreement with x-ray diffraction results, reporting the size of packing orthorhombic cells to be (4.5 nm
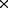
4.5 nm
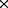
5.0 nm) and (8.06 nm
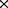
7.22 nm
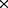
18.76 nm) for thrombin [11]–[13] and IgG Fab complexes [14], respectively.

### Fibrin Network Permeation

**Figure 3:**
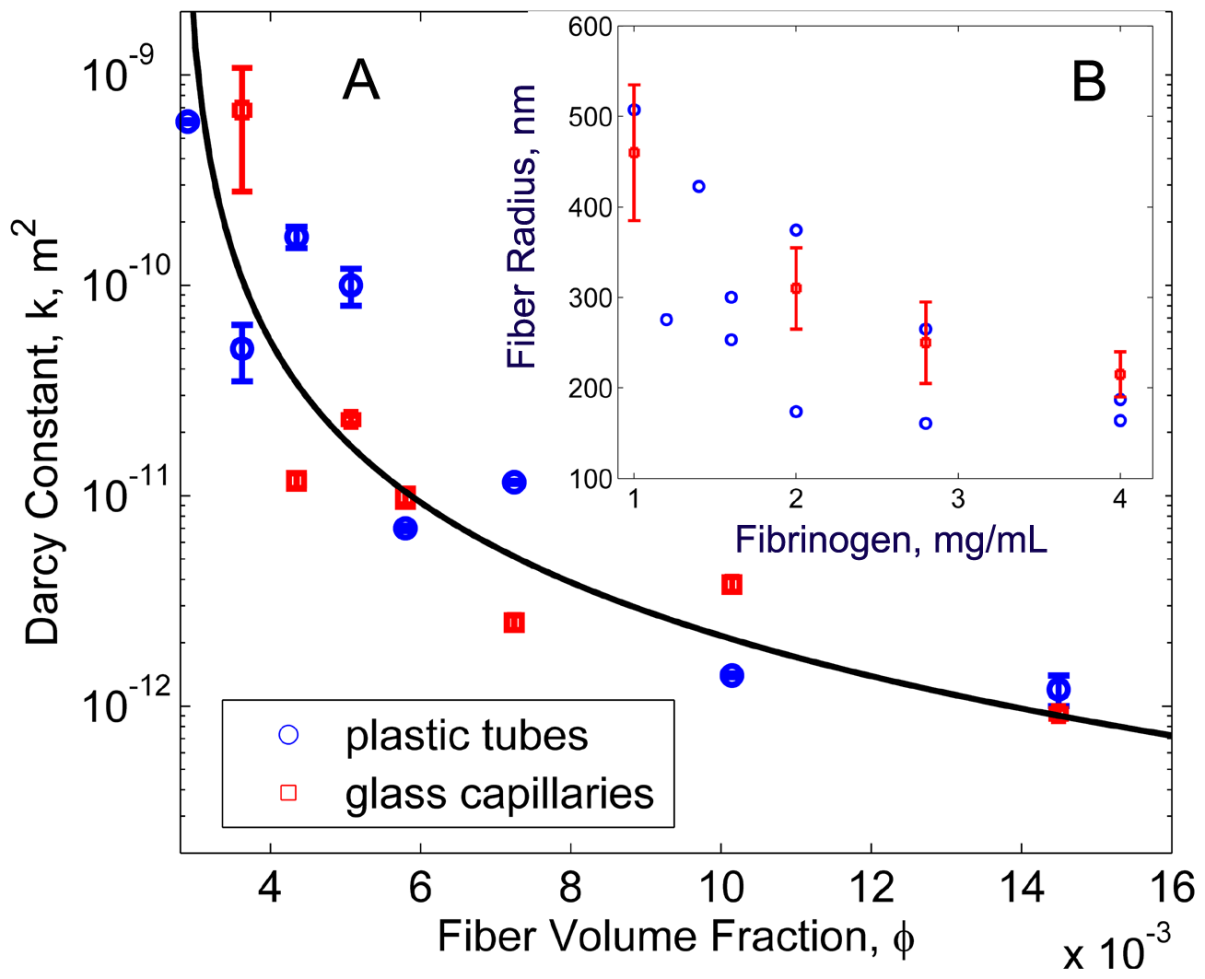
A: Permeability as a function of fiber volume fraction in fibrin gels. Circles correspond to data collected for clots in plastic tubes and squares show the data for clots in glass capillaries. Line is a fit: 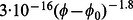, 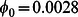. B: Fiber radius versus fibrinogen concentration determined from permeability measurements and Equation (1) (circles) and from confocal microscopy images (squares).

Permeability of the fibrin networks was quantified from Darcy's equation using liquid flow rate and pressure gradient measurements across the fibrin networks. Our measurements showed a substantial decrease of the fibrin network permeability (Darcy's constant) by three orders of magnitude, as the fibrinogen concentration increased from 0.2 mg/mL to 4 mg/mL. A power law fit was used to obtain an explicit relationship between the permeability, 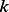, and the fibrin volume fraction, 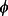. This gave 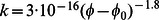, with 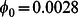 (Figure 3A). 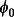 does not equal zero because it was difficult to collect consistent data for clots prepared at fibrinogen concentrations lower than 1 mg/mL. Under slight hydrostatic pressure the clot fibers deformed, broke near wall regions, and bundled together, severely disrupting the network structure. Meanwhile, clots prepared with fibrinogen concentrations higher than 1 mg/mL were stable to imposed pressure. The measured permeability 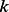 was in the range from 1 to 1000 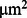. This means that the ratio of 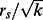, where 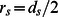 is the protein hydrodynamic radius, is much smaller than 1 and therefore, the effect of hydrodynamic hinderance on diffusion (Equation (2) in Text S2) can be neglected.

From the relation obtained by Diamond and Anand [15], [16],
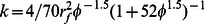
(1)the fiber radius, 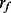, was found using the measured 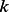 and 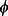 values. The calculated fiber radii ranged from 150 nm to 500 nm (Figure 3B), which is in good agreement with the fiber thicknesses evaluated directly from confocal images and with previously reported values of hydrated fiber bundles [16], [17] of coarse gels. The average fiber radius was found to decrease from approximately 450 nm to 200 nm when fibrinogen concentration increased from 1 mg/mL to 4 mg/mL.

### Protein Diffusivity in Fibrin Networks

**Figure 4:**
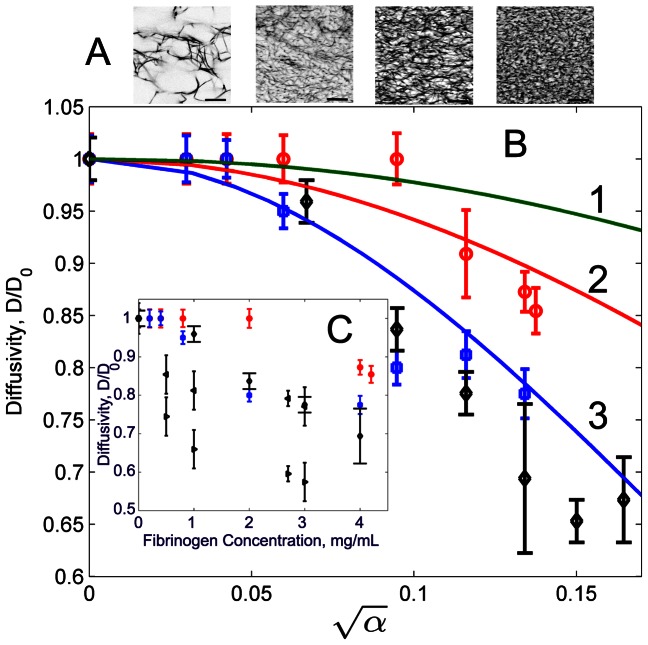
A: Confocal images of fibrin networks for different fibrinogen concentrations (left to right): 0.5 mg/mL, 2 mg/mL, 3 mg/mL, and 4 mg/mL. Scale bar is 30 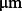. B,C: Diffusivity of different probes in fibrin gel as a function of 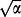 (B) and fibrinogen concentration (C): circles - thrombin molecules, squares - Fab IgG fragments, diamonds - azobenzene-labeled BSA molecules [19], left triangles - 228 nm diameter PEG-coated particles [20], right triangles - 526 nm diameter PEG-coated particles [20], 1 - Johnson's model [38] Equation (3) (Text S2); 2 and 3 - thrombin and IgG fits obtained using Equation (2). All data points refer to fibrin gels formed at 7.5 pH and thrombin concentration of 1 NIH U/mL.

The values of the diffusion coefficients and mobile fractions of thrombin and Fab IgG were obtained by fitting experimental data obtained by FRAP experiments (Text S1) using Levenberg-Marquardt curve-fitting [18]. The resulting diffusion coefficients are plotted as a function of fibrinogen concentration in Figure 4. For comparison, data reported by Stewart *et al.* 1988 [19] for BSA molecules and by Spero *et al.* 2010 [20] for 228 nm and 526 nm diameter PEG-coated particles are also shown. The diffusion coefficient of thrombin and Fab IgG in solution in the absence of fibrinogen were found to be 110
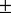
2.6 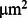/s and 40
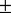
0.9 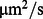, respectively. By determining the corresponding molecular diameters, one can show that these are in a very good agreement with our DLS measurements. Indeed, from the Stokes-Einstein relation 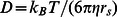, it follows that 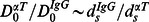. According to our FRAP measurements, the ratio of the diffusion coefficients of thrombin to that of IgG in liquid is 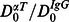 = 2.86. Taking 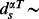
4.1 nm, yields the hydrodynamic diameter for IgG 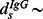
11.2 nm, which is in agreement with the value given by DLS (Figure 2B).

There are no noticeable changes in the diffusion coefficient of thrombin and Fab IgG up to a fibrinogen concentration of 2 mg/mL and 0.4 mg/mL, respectively, indicating no diffusion retardation. However, the increase of fibrinogen concentration to 4 mg/mL reduced diffusivity of Fab IgG by 22% and diffusivity of thrombin by 13%, indicating that fibrin network structure impedes the molecular diffusion due to smaller gel pore size. Pore size between fibers was estimated from microscope images to vary from 10 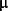
m at 0.4 mg/mL of fibrinogen to less than the microscope resolution of 0.2 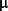
m at 4 mg/mL of fibrinogen. These estimates did not consider pores between fibrils whose dimensions were beyond the microscope resolution limit. Our results also showed that the noticeable changes of thrombin diffusivity occurred when fibrinogen exceeded 2 mg/mL. This correlates with measurements of thrombin adsorption [21], which demonstrated the thrombin absorption increase between 2 mg/mL and 4 mg/mL of fibrinogen.

Comparison of the diffusion coefficients obtained from FRAP measurements with diffusion models (Figure 4) shows that the extended Ogston's model [22] fit the data well, whereas the Johnson model given by Equation (3) in Text S2, overestimates protein diffusion. Here, the diffusivity is plotted as a function of 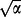, where 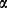 is calculated according to Equation (3). The model fitting parameters are given in Table 1.

**Table 1:** Parameters in equation (2).

Probe	Hydrodynamic diameter, 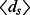 , nm	Solute flexibility, 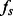	Fiber flexibility, 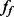	s
Fab IgG	11.2	0.1	1	27
thrombin	4.1	0.1	1	17

#### Protein Mobile Fraction

**Figure 5:**
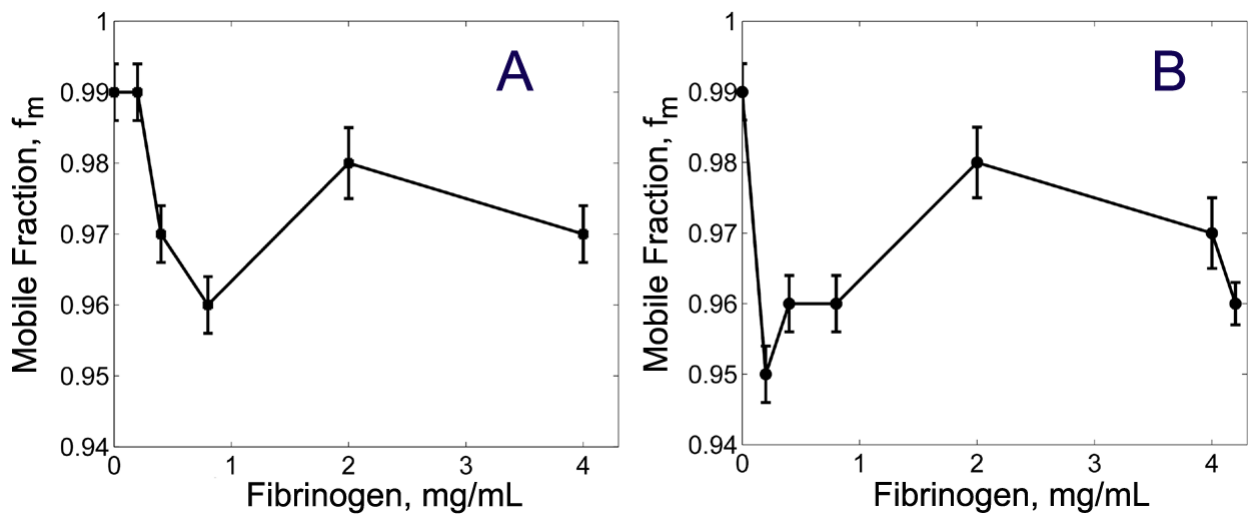
Mobile fraction of Fab IgG (A) and thrombin (B) in the fibrin gel at different fibrinogen concentrations.

From our FRAP experiments we found that a small fraction of probe molecules (1% to 5%) were immobile (Figures 5A, 5B). This suggests the importance of considering probe-fiber interaction including molecular binding and probe trapping in fibrin fiber nano-pores to understand protein transport through fibrin networks. Indeed, because fibrin polymerization occurred while probe molecules were present in the fibrinogen solution, the polymerized fibers might incorporate probe molecules. These molecules can be introduced in fibers as a solute phase, in the case of Fab IgG, or as both a solute phase and as molecules specifically bound to fibrin, in the case of thrombin. The SAXS experiments [23] revealed that depending on fibrinogen concentration, the solute phase in fibers can reach 80% with an average of 50–60%. Although gels in these experiments did not contain probe molecules, in our study, we anticipate a certain fraction of the probe proteins (non-interacting Fab IgG and possibly interacting thrombin) to be present as a solute in the fiber nano-pores. We do not expect binding of Fab IgG to the fibrin surface. However, we presume possible binding of thrombin to fibrin as demonstrated by previous studies [24], [21], in which high and low affinity sites were detected.

Surprisingly, our results show that the molecular mobility decreases not only in the dense fibrin networks, but also in the networks prepared with low (less than 1 mg/mL) fibrinogen concentrations. Clearly, the protein binding probability should be proportional to the exposed surface of the fibrin fibers. As we found (Figure 3B), the fiber thickness decreases as the fibrinogen concentration increases. Thicker fibers incorporate a larger fraction of probe molecules than thin fibers, which affects the total mobile fraction. Intuitively, as the fibrin volume fraction increases with the further increase of fibrinogen concentration (greater than 2 mg/mL), probe mobility becomes suppressed due to the larger exposed fibrin surface area and smaller pore sizes of the denser network. Our findings are consistent with [21], which indicated irreversible binding of thrombin to fibrin and dependence of binding affinity on fiber thickness. Thus, the presence of a small non-mobile fraction can be attributed to probe specific binding to fibrin fibers (thrombin) as well as probe trapping inside fibrin nano-pore structures (thrombin, IgG).

### Effect of Fibrin Cap on Hemodynamics and Protein Transport

**Figure 6:**
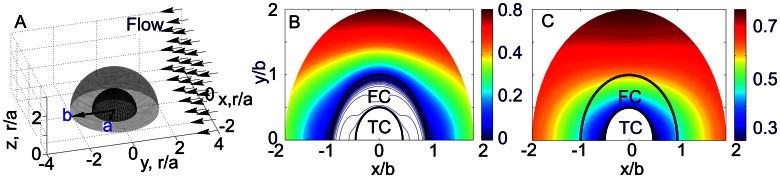
A: Model representation of a thrombus consisting of a nonpermeable core of radius 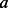 and a fibrin cap of thickness 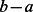, where 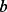 is the radius of the thrombus. Flow is uniform at infinity. B,C: Velocity field near the thrombus (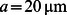, 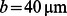) with low (B) (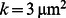) and high (C) (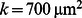) permeable fibrin cap, the flow Reynolds number, 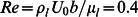. The flow at infinity is along the horizontal axis. Color scale shows the relative absolute velocity 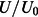.

One of the factors determining thrombus growth is the spatial distribution of hemostatic activators such as thrombin which causes activation of platelets and fibrin polymerization. Based on our observations that stabilized thrombi are covered by fibrin [2] and using our *in vitro* fibrin permeation and protein diffusivity measurements, we built a hemodynamics model to study the effect of the fibrin network on thrombin transport. In the model (Figure 6A), the geometry of a small thrombus comprises a central non-permeable core of radius 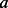 consisting of densely packed activated platelets and a permeable fibrin network covering the thrombus core. The exterior shape of a thrombus is approximated by a hemisphere of radius 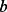. The porous structure of the fibrin cap permits blood flow within the cap, and therefore the transport of proteins includes both diffusion and advection.

To determine which mechanism drives protein transport in a thrombus and how it depends on permeability of the network and protein size we compared advective and diffusive fluxes within the fibrin cap. We calculated the flow field for a small thrombus with dimensions, 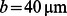 and 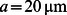 using solution for the stream function (Equations (13) and (14) in Text S3). We found that the variation of fibrin cap permeability from high to low values noticeably changes the flow through the cap. Highly permeable cap (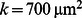) hardly affects the flow outside the thrombus core (Figure 6C). As the permeability of the cap decreases down to its minimum value of 3 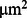, the cap performs almost as a non-permeable shell restricting flow within the thrombus (Figure 6B).

**Figure 7:**
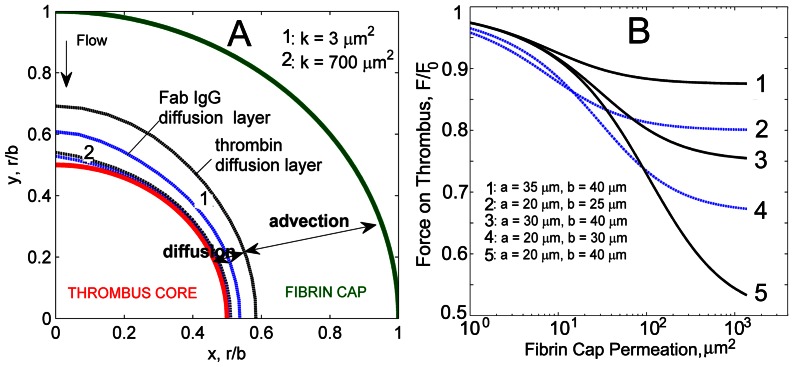
A: Diffusion and advection layers identified in the fibrin cap for thrombin and Fab IgG. Diffusion prevails near the thrombus surface and depends on molecular size. Smaller than Fab IgG complexes thrombin molecules have higher mobility resulting in thicker diffusion layer. Diffusion layer profiles for thrombin and Fab IgG for low (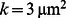) and high (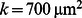) fibrin cap permeabilities are shown. Flow Reynolds number 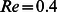, 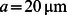, 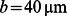. B: Force acting on a thrombus surface as a function of the permeability of the fibrin cap for different radii values of the core, 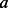, and the thrombus, 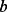. For each permeability value, the force is non-dimensionalized by the force acting on a nonpermeable thrombus of the same size.

In the vicinity of the non-permeable thrombus core, the flow velocity tends to zero according to no-slip boundary conditions, which leads to the formation of a diffusion layer near the surface of the core (Figure 7A). The thickness of this diffusion-prevailing layer can be found from the balance between advective and diffusive fluxes, 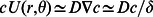, where 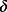 is the thickness of the diffusion layer, 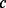 is the protein concentration on the core surface, 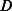 is the protein diffusivity, and 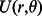 is the local advection velocity. In terms of the Peclet number, 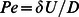, this condition means 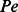 = 1, which yields the thickness of the diffusion layer profile, 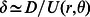. Therefore, 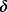 linearly depends on the protein diffusion coefficient and inversely proportional to the local advection velocity 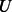, which in turn, depends on the fibrin cap permeability 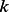 (Equation (14) in Text S3). Our calculations showed that in high permeable networks the thickness of the thrombin diffusion layer was about 4% of the thrombus radius 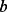, whereas for low permeable networks 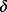 ranged from 10% to 20% of 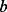. The larger Fab IgG molecules have 2x lower diffusivity resulting in a 2x thinner diffusion profile than thrombin. This points out the importance of considering effects of protein size on diffusion retardation. The presence of multiple protein molecules inside the thrombus will result in different diffusion layer profiles and therefore, different spatial distributions of proteins inside the thrombus.

#### Thrombin Transport

**Figure 8:**
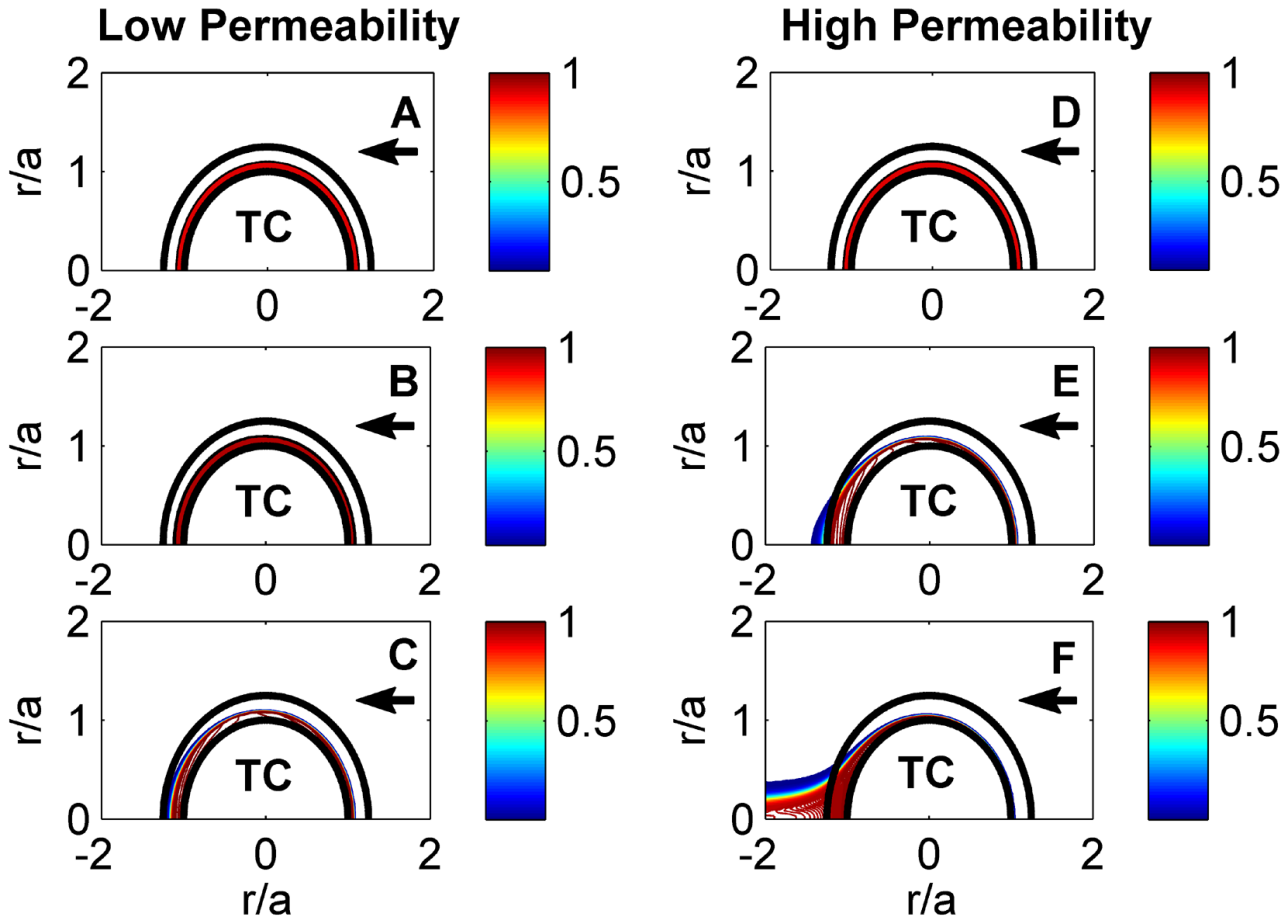
Evolution of thrombin concentration for low (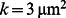) and high (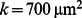) permeable fibrin networks in time: 0 s (A, D), 13 ms (B, E), 38 ms (C, F). ‘TC’ denotes a thrombus core. The arrow shows the direction of flow. The flow Reynolds number, 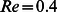. The color bar shows thrombin concentration relative to its initial value when thrombin is uniformly distributed in a 2 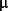
m thick layer near the thrombus core (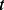 = 0 s).

To model spatial temporal evolution of thrombin, we assumed that thrombin was generated on the surface of platelets and was initially distributed in a 2 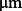 thick spherical layer near the thrombus core. The cap thickness was assumed to be 5 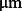 which was the thinnest cap observed in our experiments. Simulations revealed that over the time interval of 38 ms high and low permeable networks limited the transport of thrombin to various extent (Figure 8, see also Figure S2). After 38 ms thrombin appeared downstream of the high permeable thrombus cap whereas in low permeable cap thrombin just reached the surface of the cap, remaining inside the thrombus. In both cases thrombin concentration was low on most of the cap surface as the thrombin was washed downstream. These results compliment previous experimental observations [25] of platelets adhesion and aggregation downstream of a thrombus during its formation. While these observations were related to purely mechanical activation of platelets due to blood flow shear micro-gradients, our results additionally emphasize the importance of the spatial distribution of thrombin generated by the thrombus core.

#### Effect of Fibrin Cap Permeability on Thrombus Mechanical Stability

To assess the effect of fibrin cap permeability on the mechanical stability of a thrombus (embolization), the stress tensor was integrated over the thrombus surface and the hydrodynamic force 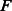 acting on the thrombus was calculated (Equation (30) in Text S3). Figure 7B shows how this force depends on the fibrin network permeability and thickness. The presence of a high permeable fibrin cap results in a force close to that acting on a thrombus core. As the fibrin volume fraction in a cap increases, 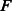 monotonically approaches the force on a non-permeable thrombus cap. Particularly, for the thrombus with dimensions of 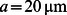 and 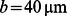, a highly permeable 20 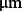 fibrin cap with 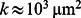, reduces 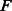 by almost 50%. Therefore, as the fibrin cap of the thrombus develops, enhancing the internal cohesive forces within the thrombus, the permeability of the cap decreases and the stress on the thrombus surface increase. As a result, if flow induces the thrombus to embolize, it is more likely that a thrombus with a denser fibrin network would detach from the vein surface as a whole.

## Discussion

This paper provides justification for a previously unrecognized possible mechanism limiting growth of small asymptomatic thrombi. The mechanism suggests that the fibrin network overlaying a thrombus observed in mice limits the thrombus growth by affecting the transport of proteins and by separating resting platelets in flowing blood from regions of high thrombin concentration generated by the thrombus core. Our analysis integrating experiments on protein diffusion and fluid permeation with the hemodynamic thrombus model revealed that permeability of the fibrin network and protein diffusivity are important factors affecting transport of blood proteins inside the thrombus.

We found that over a physiologically relevant range of fibrinogen concentrations (0.5 to 4 mg/mL) fibrin networks do not seem to form a strong diffusion barrier and that they minimally restrict the diffusion of thrombin. It was shown that the diffusivity of thrombin in the fibrin network can be hindered by 13%, whereas diffusivity of bigger molecules, Fab IgG, can be reduced by as much as 22%. We also found that the fibrin network permeability, 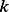, decreases by three orders of magnitude when fibrinogen concentration increases from 0.5 to 4 mg/mL. This essentially reduces advective flow in thrombi. It should be noted that although FRAP permits measuring diffusivity of proteins in a biopolymer network environment, some limitations exist when studying diffusion in extracellular matrices. As was recently shown [26], in cases where there is cellular accumulation and degradation of the protein diffusant, if diffusion is fast enough, overall FRAP kinetics tends to reflect the time scale of uptake and degradation rather than diffusion. These effects can be important when performing FRAP of thrombin diffusing through platelet aggregates composing a thrombus core. Thrombin binding to platelets' (GP) Ib-V-IX receptors [27] makes the fluorescence recovery a complex function of both binding and diffusion and therefore, requires proper quantitative interpretation [28]. In this case, one should consider Fluorescence Correlation Spectroscopy [29] as an alternative to the FRAP method to measure molecular diffusion [26].

The experimentally obtained metrics characterizing diffusive and advective transport inside the clot were incorporated into a thrombus hemodynamical model to run predictive simulations. We showed that the size of diffusive and advective regions strongly depends on the permeability, 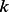, of the fibrin cap and the diffusion coefficient, 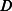, of the protein. We demonstrated that the presence of a permeable fibrin cap limits thrombin accumulation on the thrombus surface to its downstream zone. The thrombin concentration on the upstream thrombus surface and in the region above the core are insufficient to support activation of resting platelets in flowing blood near the thrombus. In a high permeable cap, advective flow washes thrombin produced on the surface of the platelets in the thrombus core downstream, thus limiting continued growth except at the very downstream surface of the thrombus. Meanwhile, a low permeable fibrin cap impenetrable to platelets can delay platelet exposure to thrombin and, subsequently, reduce platelet activation and aggregation.

The structural composition of thrombi can vary depending on the vessel type (venous versus arterial), the nature of the vessel injury, and physiological and hemodynamic conditions. Recently, [30], [31] studied thrombus development in mouse cremaster arterioles and did not observe fibrin accumulation on the luminal thrombus surface that we see on stabilized small thrombi formed in venules. There are several differences in the injury models that might account for the reported difference in thrombus structure. Arterial thrombi form at higher shear rates and are platelet rich, while venous thrombi are fibrin rich. Additionally, the injury model reported by Stalker et al [31] involves vessel rupture and clot formation in the extravascular space while in the mesentery model thrombi formed in the absence of detectable bleeding. Thus, the extent of injury and exposure of blood to the internal layers of the vessel wall is different in the two injury models. In injuries in which we induce bleeding we observe rapid fibrin accumulation at the base of the thrombus, similar to what was observed in [31].

Recently, using thrombin-sensitive platelet binding sensor (ThS-Ab), Welsh et al. [30] identified thrombin rich regions in the developing thrombus. The sensor binds CD41 on platelets and following thrombin cleavage of the ThS moiety generates a fluorophore. According to this study, the highest levels of thrombin arose between 40 and 160 seconds nearest the injury site where fibrin colocalized and where the thrombus was most mechanically stable. Low thrombin activity was detected away from the vessel wall in the thrombus and was below the minimum detection limit in the outer most, 10 micron thick, layer of the thrombus. Although thrombus composition is different in arterioles, these results are in agreement with our simulations for venous thrombi, showing that thrombin concentration decreases from the thrombus interior to its surface, down regulating thrombus growth.

Stalker et al. [31] and Voronov et al. [32] reported the transport of different size molecules through platelet-reach thrombi. It was shown [31] that greater packing density in the core facilitated contact-dependent signaling and limited entry of plasma-borne molecules visualized with fluorophores coupled to dextran and albumin. Using Lattice Boltzmann method to simulate the flow through a reconstructed thrombus and Lagrangian Scalar Tracking with Brownian motion transport to model diffusion, transport of different factors was simulated in [32]. Modelling was performed for an input average lumen blood velocity of 0.478 cm/s, which resulted in 0.2 mm/s mean flow rate within the thrombus outer shell. The shell was calculated to be 100-fold more permeable than the thrombus core with core permeability of 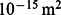. These results support our assumption that platelet core in our thrombus model can be considered impermeable relative to the fibrin cap with permeability in the range from 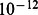 to 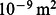.

In [32] the effective diffusion coefficients of FX, ADP and Ca were calculated and the averaged tortuosity, defined as the ratio of the diffusion coefficient in pure liquid phase to that in the clot, was found to be from 2 to 2.5. Meanwhile, our measurements provide the maximum achievable tortuosity corresponding to 4 mg/mL fibrinogen concentration to be around 1.14 for thrombin and 1.28 for Fab-IgG. Although these experimentally derived tortuosity values and tortuosity values calculated in [32] were obtained for different thrombus models and experimental conditions, it is likely the platelet clots hinder molecular diffusion to a greater extent than fibrin networks due to smaller platelet clot pore sizes. Our findings as well as results in [32] demonstrate that both fibrin cap and platelet-rich clots do not present a strong diffusion barrier for the relevant molecules, whereas, permeability can greatly affect protein transport in thrombi.

Overall, our results complement recent studies on thrombus development and emphasize the importance of spatial distributions of sites of factor activation and factor activity in regulating thrombogenesis. Our findings suggest that timely formation of a fibrin network on the surface of a developing small thrombus could limit its further growth. The proposed mechanism can prevent small blood clots from becoming large pathological thrombi creating life threatening emboli.

## Methods

### Modeling Approach

#### Biological background

Fibrin and platelets are the major components of a growing thrombus. Fibrin is formed in a thrombus when its soluble precursor protein in blood, fibrinogen, is cleaved by the enzyme thrombin and polymerizes to form a fibrin gel. Fibrin(ogen) mediates platelet adhesion by linking adjacent activated platelets and forms a network providing structural stability. Platelets are cell fragments present in blood that bind to sites of vessel injury. Upon binding to the injury site, platelets can be activated, resulting in dramatic shape changes and the release of prothrombotic factors. Activated platelets support coagulation reactions that generate thrombin to activate additional platelets and convert fibrinogen to fibrin. These activities suggest that the prothrombotic activity on the thrombus surface promotes continued growth by generating fibrin and incorporating and activating new platelets. It is not clear what processes disrupt this positive feedback mechanism and prevent thrombi from growing indefinitely [33]–[37].

Our previously developed multiscale model [4] of thrombus formation included molecular signaling considerations (platelet activation), biochemical reactions (the coagulation and anticoagulation pathways) and blood flow (hemodynamic effects). The model not only described how the thrombus developed in time but also included spatial considerations. The model predicted that platelets flowing in blood initially aggregated at the injury site, were activated by agonists and supported coagulation reactions that generated thrombin. Thrombin is not only a potent platelet activator, but also catalyzes the formation of fibrin. The model supported our experimental observations that after the formation of an activated platelet core, a fibrin network would form on the thrombus surface. It suggested that the fibrin cap forming on the thrombus surface might limit its growth by interfering with the transport of thrombin. Thus, the model integrated intracellular signaling events, a network of biochemical reactions and hemodynamic considerations to suggest a novel mechanism that might limit thrombus growth.

#### Diffusion Model

We used the extended Ogston's model [22] for thick flexible fibers, which expresses protein diffusivity in a network as follows,
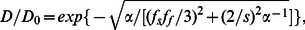
(2)where 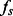 and 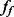 are the solute and fiber flexibility factors (see Table 1), 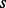 is a constant represented in a limiting step size, 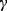, through 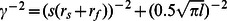, where 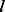 is a fiber length per unit volume, and
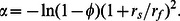
(3)For comparison, we also used the effective medium model by Johnson et al. 1996 [38] assuming multiplication of the retardation due to steric (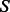) and hydrodynamic interactions (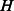) (see Text S2 for details).

#### Thrombus Hemodynamics Model

Although it was observed [39], [40] that large thrombi have very heterogenous structure, there are no direct measurements of permeability variations inside the thrombus. It was reported [9] that platelet core represents a consolidated, tightly packed mass. It was also found [9] that porosity is least at the thrombus core where platelets have undergone spreading and are tightly bound to one another and greatest in the outer regions of the thrombus. Moreover, there were some in vitro experiments [3], [41] studying the structure of fibrin networks under different conditions. These studies demonstrated small spatial variations of pore sizes within the network generated under the same conditions. Therefore, as a first order approximation, we assume that the permeability 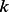 of fibrin cap is uniform and the thrombus core has a negligible permeability.

The composite sphere approach developed in [42], [43] is adopted to model the flow around and through a thrombus. The model of a thrombus employs a composite spherical structure with an impermeable core (activated platelets and fibrin) and a permeable shell (fibrin cap). Hemodynamics is modeled using Newtonian incompressible steady axisymmetric flow with velocity 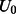 at 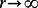. For the thrombus of radius 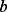 with a core of radius 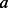, a creeping flow around the thrombus is described by the Stokes and continuity equations
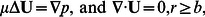
(4)where 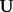 is the velocity vector (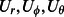), and 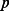 is the fluid pressure. Flow inside the fibrin cap having a uniform permeability 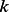 is described by the Brinkman and continuity equations
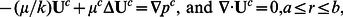
(5)where the superscript 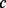 refers to macroscopic quantities in the fibrin cap region, and 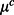 is an effective fluid viscosity in porous medium assumed to be equal to 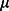. The surface of the thrombus core is assumed to be nonpermeable and no-slip boundary conditions are imposed at 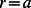. On the surface of the fibrin cap (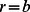) the continuity of the velocity vector and the stress tensor are stated and a uniform flow is imposed at 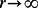 (see Text S3 for details).

Transport of thrombin is modeled by the diffusion-advection equation coupled with Equations (4) and (5)

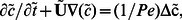
(6)where the flow velocity and thrombin concentration are non-dimensionalized as, 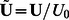, 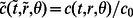. Coordinates and time are nondimensionalized as 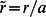 and 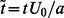, where 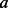 is the radius of the thrombus core and 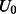 is the flow velocity at 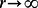. The Peclet number is defined as 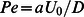, and 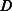 is the diffusion coefficient of thrombin determined from our experiments. Equation (6) is solved numerically using minmod flux limiter method [44] with the following boundary conditions in the interval 0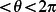: 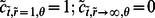, and the initial distribution of thrombin in the form of a spherical layer near the thrombus core: 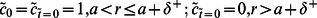, where 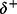 is the thickness of the initial thrombin layer.

It should be noted that the use of Stokes and Brinkman-Darcy equations is valid if the local Reynolds number is less than 1. At higher Reynolds numbers inertial effects can be significant and the relation between liquid flow rate and pressure gradient is no longer linear. However, the local Reynolds number can be small even if the Reynolds number calculated with respect to the blood vessel diameter is large. Assuming a parabolic flow velocity profile in a blood vessel of radius 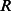 and a small thrombus (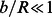), it is straight-forward to show that the criterion, 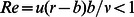, where 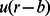 is the velocity of the flow in the vicinity of the thrombus and 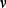 is the blood viscosity, yields 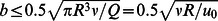. Here, 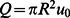, is the volumetric flow rate through the vessel, 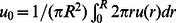 is the axial averaged blood flow velocity, and 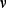 is the blood viscosity. Assuming a vessel radius of 200 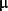
m, an average velocity of 1 cm/s, and a blood viscosity of 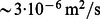, the model limitation on the thrombus size is 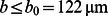. In experiments, the observed thrombi were smaller than 60 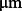 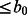, and therefore the use of Equations (4) and (5) is justified.

## Materials and Methods

### 

#### Fibrin gel formation

Fibrinogen (Enzyme Research Laboratory, South Bend, IN) was aliquoted in 30 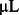 at 20 mg/mL and stored at −80°C for later use. Desiccated labeled fibrinogen (5 mg) (Life Technologies, Grand Island, NY) was dissolved in 20 mM sodium citrate at pH 7.4. The preparation was frozen at −80°C in 5 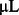 aliquots at 4 mg/mL. Human 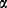-thrombin (Enzyme Research Laboratory, South Bend, IN) was dissolved in 50 mM sodium citrate, 0.2 M NaCl, 0.1% PEG-8000 at pH 6.5 and stored at −80°C as 3 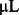 aliquots at 1 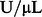. Aliquots were thawed only once at 37°C before each experiment. Labeled thrombin was generated by incubating fluorescein labeled thrombin inhibitor FPR-chloromethylketone (PPACK) (Hematologic Technologies, Essex Junction, VT) with 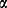-thrombin (Enzyme Research Labs, South Bend, IN) in PBS buffer. Labeled thrombin was purified from unbound fluorescein-PPACK by gel filtration, aliquoted and stored at −20°C. The labeled Fab fragment (AlexaFlour 488 goat anti mouse IgG Fab, 2 mg/mL) was purchased from Life Technologies (Grand Island, NY). Gels were prepared by first, mixing fibrinogen, labeled fibrinogen, and probe molecules with buffer in a tube. The ratio of unlabeled to labeled fibrinogen was 1∶20 and the final fibrinogen concentration ranged from 0.2 to 4.2 mg/mL. The Fab Igg concentration was 0.02 mg/mL and labeled thrombin concentration was 0.01 mg/mL. Polymerization was initiated by adding thrombin (1 U/mL final), the solution was rapidly pipeted 7–8 times and injected into glass microchannels (VitroCom, NJ) of rectangular cross sections (200 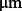
×2 mm, 3 cm long, wall thickness 20 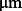). Microchannels were placed on a wet paper tissue inside a sealed petri dishes and gels were incubated for 3 hours for a complete fibrinogen polymerization. For FRAP, the ends of the channels were then sealed with silicon grease to prevent evaporation and eliminate any internal flow.

The volume fraction of fibrin, 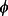, in the prepared gels was determined as
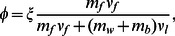
(7)where 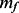, 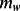, and 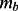 are masses of desiccated fibrinogen, water, and buffer and 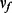, 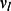 are the specific volumes of fibrinogen (0.725 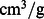), and water-buffer solution (1.02 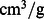), respectively. Masses 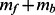 and 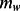 were determined from weighing of hydrated and dehydrated gel and 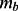 was known from the preparation protocol, 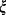 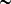
5 is a factor accounting for the difference between fibrin and fibrinogen densities [23], [45].

#### Clot permeability measurements

To measure permeability, clots were prepared in transparent plastic tubes and glass capillaries having internal diameter of 1.5 mm and 1 mm, respectively. The length of plastic tubes was 2 cm and the length of glass capillaries varied from 1 cm to 1.9 cm. Distilled water was perfused hydrostatically by gravity through the tube. The flow rate was determined by measuring the mass of outflow liquid with scales (Acculab, ALC-320.3, 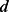 = 0.001 g) at different times for three different pressure drops. The pressure drop across the gel was measured using a pressure transducer as well as by liquid column height measurements. Permeability was determined from Darcy's equation as 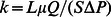, where 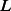 is the length of the cylindrical clot, 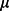 is liquid viscosity, and 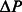 is the pressure drop across the clot.

#### Protein size measurements

Dynamic light scattering (DLS) was used to measure thrombin and Fab IgG hydrodynamic diameters. In DLS, Brownian motion is measured and related to the size of the particles through the Stokes-Einstein equation 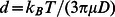, where 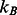 is the Boltzmann's constant, 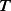 is the temperature, 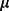 is the dynamic viscosity, and 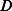 is the diffusion coefficient. Since most molecules are not spherical, the hydrodynamic diameter is determined as the diameter of a sphere that has the same translational diffusion speed as the molecule. Measurements were performed at 30°C in three series of 15 measurements for each sample of thrombin and Fab IgG solutions. Zetasizer Nano S90 with Zetasizer software was used for DLS data collection and analysis.

#### Microscope equipment

FRAP measurements and fibrin network imaging were performed using laser confocal microscope Zeiss LSM 510 Meta with 20×, 63×, and 100× objectives (20/0.4, 63x/1.4 Oil DICIII, and 100x/1.4 Oil DICIII). The sample ROI was bleached using 720 nm laser line (Multiphoton Coherent Chameleon XR tunable pulsed laser, 705–980 nm, maximum power 1 W). The bleaching was performed in circular ROIs at typical laser output power of 25–50 mW. For monitoring a recovery signal from labeled thrombin and Fab Igg, the sample was illuminated using the 514 nm laser line from an Argon-ion laser. The emitted fluorescence was detected after passing through 544–608 nm band pass filter. For acquisition of fibrin network images, the pinhole size was adjusted to 1 Airy unit and a 561 nm laser line (DPSS561-10) with 544–608 nm band pass filter were used. The output laser power during image acquisition ranged from 0.1 to 0.15 mW. Details of FRAP method are provided in Text S1.

## Supporting Information

Figure S1
**Force on a thrombus as a function of non-dimensional fibrin cap thickness** 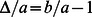 **for different values of the parameter** 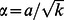 **. A:** 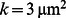 **, B:** 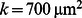 **.**
(TIF)

Figure S2
**Simulation results of thrombin distribution for (a) low (** 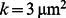 **) and (b) high (** 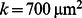 **) permeable fibrin networks after 75 ms of being exposed to external flow,** 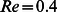 **. ‘TC’ and ‘FC’ denote a thrombus core and a fibrin cap.**
(TIF)

Text S1 **FRAP method.**
(PDF)

Text S2 **Diffusion model.**
(PDF)

Text S3 **Thrombus hemodynamics model.**
(PDF)
