# Supplementary figures and images for: Fibrin Networks Regulate Protein Transport during Thrombus Development
Source: PLoS Comput Biol. 2013 Jun 13;9(6):e1003095. doi: 10.1371/journal.pcbi.1003095 (PMC3681659; doi:10.1371/journal.pcbi.1003095)

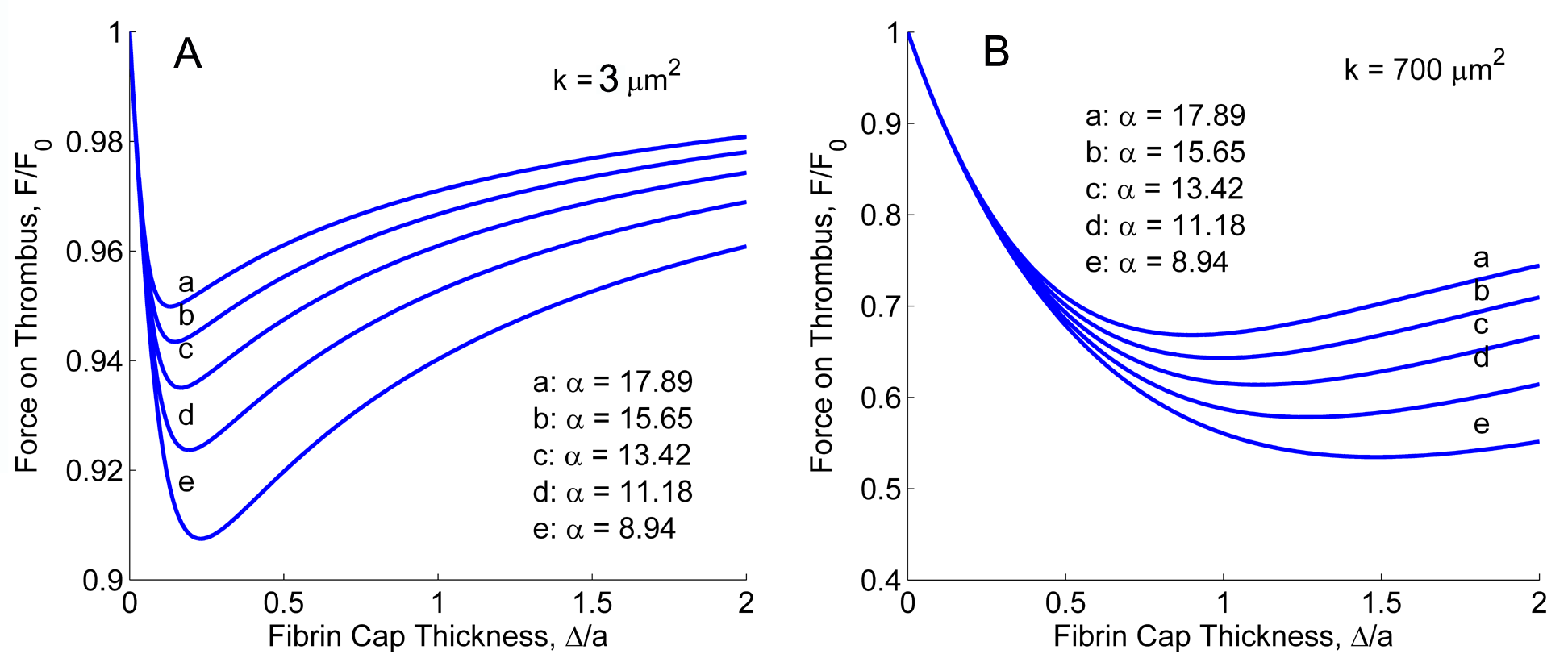

Supplement: Figure S1 — Force on a thrombus as a function of non-dimensional fibrin cap thickness for different values of the parameter . A: , B: . (TIF) [file pcbi.1003095.s001.tif]

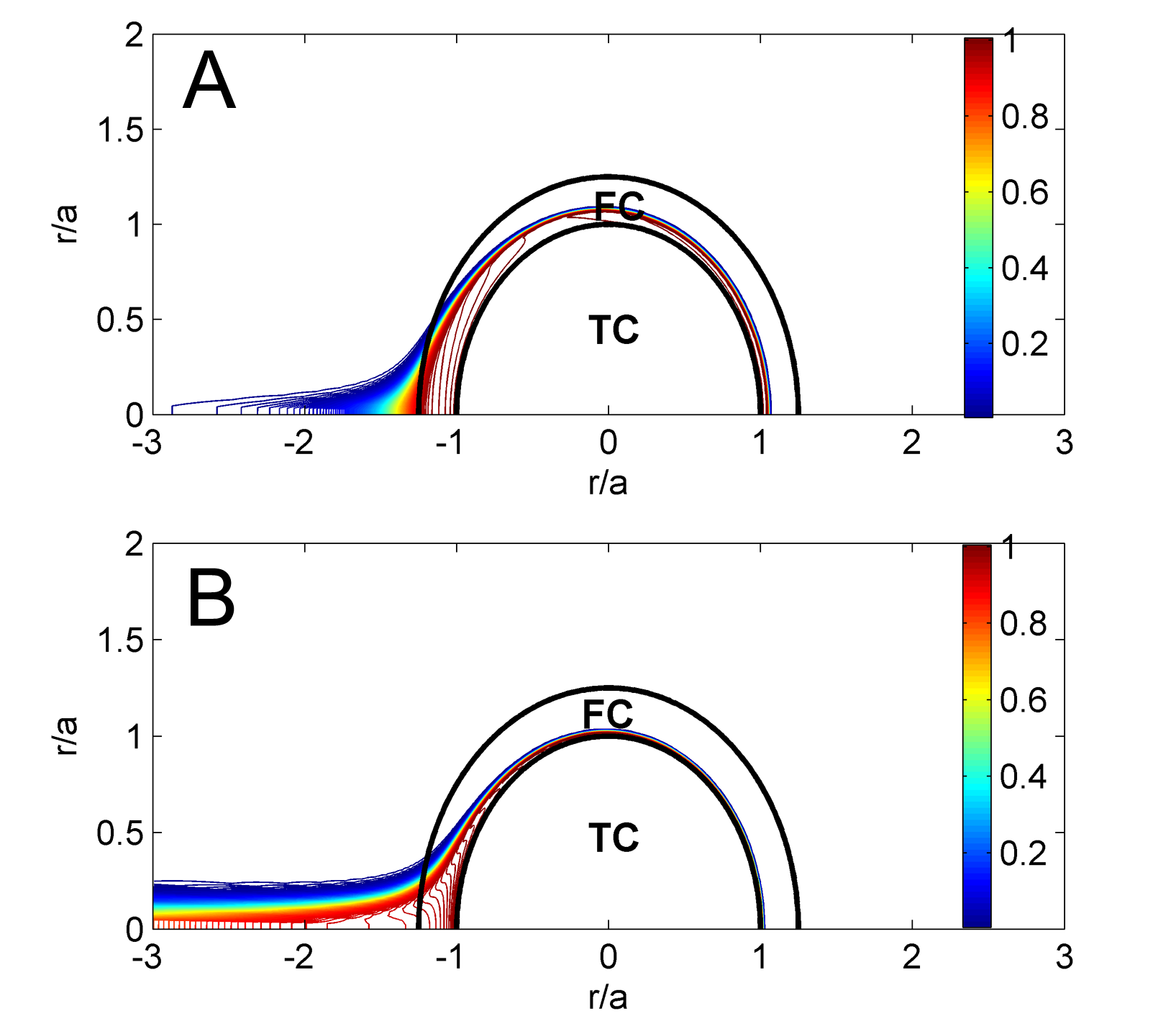

Supplement: Figure S2 — Simulation results of thrombin distribution for (a) low ( ) and (b) high ( ) permeable fibrin networks after 75 ms of being exposed to external flow, . ‘TC’ and ‘FC’ denote a thrombus core and a fibrin cap. (TIF) [file pcbi.1003095.s002.tif]
