# Supplementary material for: Fibrin Networks Regulate Protein Transport during Thrombus Development
Source: PLoS Comput Biol. 2013 Jun 13;9(6):e1003095. doi: 10.1371/journal.pcbi.1003095 (PMC3681659; doi:10.1371/journal.pcbi.1003095)
Supplement: Text S1 — FRAP method. (PDF) [file pcbi.1003095.s003.pdf]

Text S1

FRAP Method

The technique of fluorescence recovery after photobleaching (FRAP) is used for quantifying the mobility of molecules and particles on a microscopic level. In FRAP experiments molecules of interest are tagged with fluorescent labels. Following the saving of a background before photobleaching, the sample region of interest (ROI) is subjected to intense laser radiation which causes the fluorescence lifetime of fluorophores to quickly elapse. The fluorescence signal recovers as unbleached probes from surrounding regions diffuse into bleached region. The fluorescence recovery curve is used to determine the diffusion coefficient by solving diffusion equation in ROI using the known light intensity distribution (point-spread-function (PSF)) [1].

Assuming a Gaussian distribution in radial and axial directions, the bleaching PSF is given by

$$I_b(x,y,z) = I_{0b}\exp\left[-2(x^2+y^2)/r_0^2\right]\exp\left(-z^2/z_0^2\right), \tag{1}$$

where r_0 and z_0 are the radial and axial resolution values. The distribution of the chromophores after bleaching of a uniform disk can be expressed as follows,

$$\begin{aligned} C(r,z) &= C_0(r,z)\exp\left(K_0\right)\exp\left[-2(z/z_0)^2\right], r \leq r_d \\ C(r,z) &= C_0, r > r_d \end{aligned} \tag{2}$$

where K_0 is a bleaching parameter.

The total fluorescence intensity inside the bleaching disk can be calculated [1] as follows,

$$\frac{F_{tot}}{F_0} = 1 + \frac{1}{\mathrm{erf}(\sqrt{2}\Delta z_0/z_0)}\sum_{n=1}^{\infty}\left[\frac{-K_0^n}{n!\sqrt{\alpha_n}}\mathrm{erf}\left(\sqrt{\frac{2\alpha_n}{\alpha_n - n}}\frac{\Delta z_0}{z_0}\right)\right] L(t) , \tag{3}$$

where $L(t) = (1 - e^{-2\tau_r/t}(I_0(2\tau_r/t) + I_1(2\tau_r/t)))$, F_0 is the total fluorescence intensity inside the disk before bleaching, Δz_0 accounts for finite pinhole aperture by rejecting the light from regions above δz_0 and below $-\delta z_0$, I_0 and I_1 are the modified Bessel functions of the zeroth and of the first orders, K_0 is a bleaching parameter. Also, $\alpha_n = 1 + n(1 + 2\frac{t}{\tau_z})$, $\tau_r = w^2/(4D)$ and $\tau_z = z_0^2/(4D)$ are the corresponding radial and axial characteristic diffusion times. For a fully opened aperture, Equation (3) becomes

$$\frac{F_{tot}}{F_0} = 1 + \sum_{n=1}^{\infty}\left[\frac{-K_0^n}{n!\sqrt{\alpha_n}}\frac{\Delta z_0}{z_0}\right] L(t). \tag{4}$$

In the case of using objectives with low numerical apertures (NA) ($z_0 \to \infty$), this yields

$$\frac{F_{tot}}{F_0} = 1 + P = 1 + \sum_{n=1}^{\infty}\left[\frac{-K_0^n}{n!\sqrt{1+n}}\right] L(t). \tag{5}$$

This equation is equivalent to that derived by Soumpasis [2] for the 2D diffusion in a disk of uniform radial intensity distribution. In our present work, we use lenses with relatively low NA = 0.4 and, therefore, Equation 5 can be used. It should be noted that at $t = 0$ numerical solution of Equation (5) does not exist but can be calculated by using its asymptotic value as $t \to 0$

$$\frac{F_{tot}}{F_0} = 1 + \sum_{n=1}^{\infty}\left[\frac{-K_0^n}{n!\sqrt{1+n}}\right]. \tag{6}$$

If f_m is the molecular fraction that is replenished by diffusion (mobile fraction), then Equation (5) has a modified form

$$\frac{F_{tot}}{F_0} = 1 + f_m P. \tag{7}$$

Fluorescence recovery curves were obtained from the total intensity fluorescence values in the ROI for each frame of the temporal sequence. The curves were then corrected for background and laser scanning bleaching, and normalized to the initial value.

References

1. Braeckmans K, Peeters L, Sanders N, De Smedt S, Demeester J (2003) Three-dimensional fluorescence recovery after photobleaching with the confocal scanning laser microscope. Biophysical journal 85: 2240–2252.

2. Soumpasis D (1983) Theoretical analysis of fluorescence photobleaching recovery experiments. Biophysical journal 41: 95–97.
