# Supplementary material for: Fibrin Networks Regulate Protein Transport during Thrombus Development
Source: PLoS Comput Biol. 2013 Jun 13;9(6):e1003095. doi: 10.1371/journal.pcbi.1003095 (PMC3681659; doi:10.1371/journal.pcbi.1003095)
Supplement: Text S2 — Diffusion model. (PDF) [file pcbi.1003095.s004.pdf]

Text S2

Diffusion Model

Different models have been developed to describe restricted diffusion of molecules in fibrous structures [1, 2]. Among them, Ogston's model [1] and effective medium model [2] are the most popular ones. Ogston's diffusion model accounts for fiber and solute radii and fiber volume fraction, while the effective medium model uses solute radius and gel permeability. According to Ogston's model, diffusivity in the network of randomly oriented, infinitely thin and infinitely long fibers is given by

$$D/D_0 = e^{-\sqrt{\alpha}}, \tag{1}$$

where $\alpha = \pi r_s^2 l$, r_s is a solute radius, and l is a fiber length per unit volume. The effective field model suggests that the diffusivity of solute is hindered due to hydrodynamic friction by fibers [2] ,

$$D/D_0 = \left[1 + \sqrt{r_s^2/k} + r_s^2/(3k)\right]^{-1}, \tag{2}$$

where k is the permeability of the gel. Johnson et al. 1996 [3] suggested that retardation due to steric (S) and hydrodynamic interactions (H) should be multiplied. The ratio of diffusivity in the fiber gel to that in solution is given by

$$D/D_0 = H \times S = e^{-a\phi^b} e^{-0.84 f^{1.09}}. \tag{3}$$

Here H and S are the stretched exponential functions derived by Clague and Phillips [4] and Johansson and Lofroth [5], respectively. The adjusted volume fraction, $f = \phi(1+\lambda)^2$, is expressed in terms of the fiber volume fraction ϕ and the ratio of the solute diameter to fiber radius $\lambda = r_s/r_f$. Values for a and b were corrected by Amsden [6], $a = \pi$, $b = 0.174 ln(59.6/\lambda)$.

References

1. Ogston A, Preston B, Wells J (1973) On the transport of compact particles through solutions of chain-polymers. Proceedings of the Royal Society of London A Mathematical and Physical Sciences 333: 297.

2. Phillips R, Deen W, Brady J (1989) Hindered transport of spherical macromolecules in fibrous membranes and gels. AIChE journal 35: 1761–1769.

3. Johnson E, Berk D, Jain R, Deen W (1996) Hindered diffusion in agarose gels: test of effective medium model. Biophysical journal 70: 1017–1023.

4. Clague D, Phillips R (1997) A numerical calculation of the hydraulic permeability of three-dimensional disordered fibrous media. Physics of Fluids 9: 1562.

5. Johansson L, Lofroth J (1991) Diffusion and interaction in gels and solutions: I. method. Journal of colloid and interface science 142: 116–120.

6. Amsden B (1998) Solute diffusion in hydrogels.:: An examination of the retardation effect. Polymer Gels and Networks 6: 13–43.
