# Supplementary material for: Fibrin Networks Regulate Protein Transport during Thrombus Development
Source: PLoS Comput Biol. 2013 Jun 13;9(6):e1003095. doi: 10.1371/journal.pcbi.1003095 (PMC3681659; doi:10.1371/journal.pcbi.1003095)
Supplement: Text S3 — Thrombus hemodynamics model. (PDF) [file pcbi.1003095.s005.pdf]

# Text S3

## Thrombus Hemodynamics Model

For $0 \leq \theta < 2\pi$, we use the following boundary conditions on the thrombus surface and on the thrombus core,

$$
\begin{aligned}
U_r^c(a,\theta) &= 0 & (1)\\
U_\theta^c(a,\theta) &= 0 & (2)\\
U_r(b,\theta) &= U_r^c(b,\theta) & (3)\\
U_\theta(b,\theta) &= U_\theta^c(b,\theta) & (4)\\
\tau_{rr}(b,\theta) &= \tau_{rr}^c(b,\theta) & (5)\\
\tau_{r\theta}(b,\theta) &= \tau_{r\theta}^c(b,\theta) & (6)\\
U_r(r\to\infty,\theta) &= -U_0\cos(\theta) & (7)\\
U_\theta(r\to\infty,\theta) &= U_0\sin(\theta), & (8)\\
& & (9)
\end{aligned}
$$

where $\tau_{rr} = p - 2\mu\frac{\partial U_r}{\partial r}$, and $\tau_{r\theta} = -\mu\left[(1/r)\frac{\partial U_r}{\partial \theta} + r\frac{\partial(U_\theta/r)}{\partial r}\right]$ are the normal and tangential components of the stress tensor. For the axisymmetric case, the problem can be reformulated in terms of stream function, which for the Stokes and Brinkman equations yields:

$$H^2\psi = 0, r \leq b, \tag{10}$$

and

$$H^2\psi^c - \frac{1}{k}(\mu/\mu^c)H\psi = 0, a \leq r \leq b, \tag{11}$$

where operator $H$ is expressed in spherical coordinates as follows

$$H = \frac{\partial^2}{\partial r^2} + \frac{\sin\theta}{r^2}\frac{\partial}{\partial\theta}\left(\frac{1}{\sin\theta}\frac{\partial}{\partial\theta}\right). \tag{12}$$

The general solution of Equations (10, 11) is given by [1]

$$\psi(\eta,\theta) = -0.5kU_0\left[C_1/\eta + C_2\eta + C_3\eta^2 + C_4\eta^4\right]\sin^2(\theta), \eta \geq \beta, \tag{13}$$

$$\psi^c(\eta,\theta) = -0.5kU_0[K_1 + K_2]\sin^2\theta, \alpha \leq \eta \leq \beta, \tag{14}$$

$$K_1 = C_5/\eta + C_6\eta^2 + C_7\left(\frac{\cosh\eta}{\eta} - \sinh\eta\right), \tag{15}$$

$$K_2 = C_8\left(\frac{\sinh\eta}{\eta} - \cosh\eta\right), \tag{16}$$

where $\eta = r/\sqrt{k}$, $\alpha = a/\sqrt{k}$, $\beta = b/\sqrt{k}$. Coefficients $C_1$ - $C_8$ in Equations (13, 14) are determined from the boundary conditions (2-9) and are equal to [1]:

$$C_1 = \beta^3 - \beta^2C_2 + C_5 + \beta^3C_6 + (\cosh\beta - \beta\sinh\beta)C_7 + (\sinh\beta - \beta\cosh\beta)C_8, \tag{17}$$

$$C_2 = 0.5\frac{C_0}{\alpha\sinh\beta - \cosh\alpha}(1/A), \tag{18}$$

where

$$C_0 = 3(\alpha^4 + 2\alpha\beta^3 + 3\alpha^2)\cosh\alpha + 9\alpha^2(\cosh\beta - \beta\sinh\beta - \alpha\sinh\alpha) + 3\cosh\Delta\left[(\alpha^3 + 2\beta^3 + 3\alpha)(\alpha\beta\sinh\beta - \alpha\cosh\beta - \beta\cosh\alpha) + 3\alpha^2\beta\sinh\alpha\right] + 3\sinh\Delta\left[(\alpha^3 + 2\beta^3 + 3\alpha)\cosh\alpha + 3\alpha^2(\alpha\beta\sinh\beta - \alpha\cosh\beta - \sinh\alpha)\right], \quad (19)$$

$$A = -6\alpha + (3\alpha + 3\beta + \alpha^3 + 2\beta^3)\cosh\Delta + 3(\alpha^2 - 1)\sinh\Delta, \quad (20)$$

with $\Delta = \beta - \alpha$.

$$C_3 = -1, \quad (21)$$

$$C_4 = 0, \quad (22)$$

$$C_5 = 2C_2 + 2\beta^3 C_6, \quad (23)$$

$$C_6 = (C_7\cosh\alpha + C_8\sinh\alpha)/(3\alpha), \quad (24)$$

$$C_7 = \frac{3\alpha - (\alpha\cosh\beta - \sinh\alpha)C_8}{\alpha\sinh\beta - \cosh\alpha}, \quad (25)$$

$$C_8 = (1/A)\left[3(\alpha^2 + 2\beta^3)\cosh\alpha + 9\alpha(\cosh\alpha - \alpha\sinh\alpha - \cosh\beta + \beta\sinh\beta)\right], \quad (26)$$

The components of the stress tensor are given by

$$\tau_{r\theta} = kU_0\mu\sin\theta\left(\frac{C_1\sqrt{k}}{r^4} + \frac{1}{r}\frac{C_3}{k}\right), r \geq b, \quad (27)$$

$$\tau_{rr} = p + 2\mu kU_0\cos\theta\left(3C_1\sqrt{k}/r^4\right), r \geq b. \quad (28)$$

The drag force on the thrombus is determined by integrating the stress components over the thrombus surface,

$$F = \pi b^2\int_0^\pi(\tau_{rr}\cos\theta - \tau_{r\theta}\sin\theta)_{r=b}\sin\theta d\theta, \quad (29)$$

which yields,

$$F = 2\pi b\mu U_0\Lambda_1, \quad (30)$$

with $\Lambda_1 = C_2/\beta$.

# References

1. Masliyah J, Neale G, Malysa K, GM Van De Ven T (1987) Creeping flow over a composite sphere: solid core with porous shell. Chemical engineering science 42: 245–253.
